# Enhancing employee performance through motivation: the mediating roles of green work environments and engagement in Jakarta’s logistics sector

**DOI:** 10.3389/fsoc.2024.1392229

**Published:** 2024-05-13

**Authors:** Dewi Nusraningrum, Aisyah Rahmawati, Walton Wider, Leilei Jiang, Lester Naces Udang

**Affiliations:** ^1^Faculty of Economics and Business, Universitas Mercu Buana, Jakarta, Indonesia; ^2^Trisakti Institute of Transportation and Logistics, Jakarta, Indonesia; ^3^Faculty of Business and Communications, INTI International University, Nilai, Negeri Sembilan, Malaysia; ^4^Faculty of Education and Liberal Arts, INTI International University, Nilai, Negeri Sembilan, Malaysia; ^5^Faculty of Liberal Arts, Shinawatra University, Pathum Thani, Thailand; ^6^Educational Psychology, College of Education, University of the Philippines, Diliman, Philippines

**Keywords:** employee performance, motivation, green work environment, employee engagement, logistic company, employment policy

## Abstract

This study aims to analyze the mediating role of employee engagement and the green work environment in the relationship between motivation and the performance of logistics company employees in Jakarta, Indonesia. Employing a causal quantitative research approach, we distributed 222 questionnaires among logistics employees from four surrounding cities in Jakarta, namely Bogor, Depok, Tangerang, and Bekasi. These questionnaires were adapted from past studies. The data were processed using Structural Equation Modeling (SEM) with Partial Least Squares. The results showed that employee performance in logistics companies was positively and significantly influenced by motivation. Furthermore, a green work environment and employee engagement were found to significantly mediate the relationship between motivation and performance. These findings underscore the importance of a green work environment and employee engagement in enhancing motivation and performance in logistics companies. The study implies that employee performance in logistics companies can be elevated through the provision of a green work environment, alongside fostering employee motivation and engagement.

## Introduction

The logistics market is highly competitive, and all organizations must gain and retain market shares through effective human resource management practices, which are essential in maximizing employee performance to achieve goals (Noe et al., 2019). In Jakarta, 4,836,977 employees work in various companies (Putra, 2020), supporting their organizations in realizing goals and objectives. Numerous studies have found that motivation positively affects employee performance (Danish et al., 2015; Li et al., 2015; Bergström and Martínez, 2016; Siswanto et al., 2019). Another key factor influencing employee performance is the work environment. The interaction of employees within this environment is crucial in fulfilling their duties and obligations. A comfortable work environment fosters smooth interpersonal relationships and communication among team members, thereby maximizing performance. Conversely, a chaotic environment with unfair competition can lead to employee burnout and reduced productivity (Kurniawaty et al., 2019).

The role of human resource management in motivation and work engagement is becoming increasingly important (Cimbaljević et al., 2020). There is a clear influence of motivation on employee performance; higher motivation correlates with higher performance (Yousaf et al., 2015; Paais and Pattiruhu, 2020; Qomariah et al., 2022; Royani et al., 2022; Taryana et al., 2023). Every company strives to retain its employees; thus, companies must attend to their employees’ needs and welfare. Neglect such as inadequate attention, low wages, and an unhelpful work environment can result in reduced productivity, which is the efficiency of employees in converting inputs into useful outputs (Raziq and Maulabakhsh, 2015). Higher productivity means producing more, and of better quality, with the same effort (Nusraningrum, 2018). A decline in productivity can be attributed to several factors, including a lack of work motivation. This lack of motivation can lead to diminished awareness of productivity among employees (Paais and Pattiruhu, 2020). Additionally, an unsupportive work environment can also lead to suboptimal and declining productivity (Shahzad et al., 2023).

The debate regarding how motivation can enhance employee performance is deeply rooted in the theory of human behavior, which encompasses both intrinsic and extrinsic motivation. It is often argued that the type of motivation driving individuals to work is somewhat irrelevant. Empirical evidence suggests that employees who are satisfied with their jobs are not necessarily more productive (Frey, 1997). Recent research has integrated green work environment variables with employee involvement to produce green performance in hotels, revealing that organizational leaders who are highly committed to motivation can achieve the performance expected by the company (Karatepe et al., 2022). Implementing a sustainable organization has become a business strategy, where motivating employees to engage in environmentally friendly operations does not compromise quality and efficiency (Shulla et al., 2020; Nusraningrum et al., 2023a).

Given the facts and literature reviewed, researchers are interested in surveying logistics employees in Jakarta, Bogor, Depok, Tangerang, and Bekasi (Jabodetabek) to study the influence of motivation, work environment, and employee engagement on performance. A preliminary survey of 30 logistics employees in Jabodetabek indicated that 90% of respondents observed performance issues in logistics companies, with employees unable to complete tasks on time. Additionally, 85% or more of the respondents feel that employees do not enjoy their jobs. Regarding the work environment, 90% of respondents disagreed that it is comfortable and well-equipped. There is a notable issue with employee engagement, with 95% of employees not being engaged in their work, and often unable to work continuously for extended periods. Based on the literature review and survey results, the researcher decided to further analyze the issues of motivation, work environment, employee engagement, and employee performance in Jabodetabek (Greater Jakarta), Indonesia.

## Literature review

### Employee performance

Employee performance is defined as the effectiveness and efficiency with which individuals carry out their core duties and responsibilities as outlined in their formal job description. It represents an essential human contribution to the organization (Robbins and Judge, 2017). Employee performance includes activities and results that align with organizational goals and are critical for gaining a competitive advantage. Therefore, Human Resources Management decisions seek to improve this aspect (Noe et al., 2019; Daito et al., 2020). Brumback (1988) defines performance as both behavior and outcome, in which mental and physical effort is applied to a task, resulting in action (Armstrong and Taylor, 2020). Employee Performance is intended to align with the organization’s functions, tasks, and goals (Nusraningrum, 2012). Individual performance is determined by the multiplication of three factors: individual ability, organizational support, and effort, and it suffers when one or more of these factors are missing or reduced. These include skills, interests, and personality traits (abilities); training, equipment, performance standards, management, and coworkers (organizational support); and motivation, work ethic, attendance, and job design (effort).

Employee performance dimensions include outcomes, behaviors, and traits (Mathis et al., 2017). The outcomes dimension is concerned with the quality and quantity of work produced, as well as the effectiveness and efficiency with which an employee performs. The behavior dimension takes into account factors such as adherence to work schedules, communication with superiors, attitude at work, and teamwork. The traits dimension evaluates an employee’s character traits, such as their initiative in beginning new tasks and their ability to contribute useful ideas to the organization. This study’s goal is to comprehensively measure employee performance across these three key dimensions that capture various aspects of an individual’s contribution to the organization. By evaluating outcomes, behaviors, and traits, the study provides a comprehensive assessment of employee performance, which is critical for understanding and improving individual and organizational success.

### Motivation

Motivation is the drive that maintains the intensity, direction, and persistence of an individual’s energy towards goals. Motivated employees typically perform better at their jobs (Robbins and Judge, 2017). Early theories include Maslow’s hierarchy of needs and Herzberg’s motivation/hygiene theory. Maslow suggests an ascending order of needs from physiological to self-actualization, while Herzberg posits that motivators (intrinsic motivation) and hygiene factors (extrinsic motivation) have different impacts on motivation. Self-Determination Theory (STD), proposed by Ryan and Deci, divides motivation into intrinsic and extrinsic categories. Intrinsic motivation is driven by inherent interest, while extrinsic motivation seeks to achieve separable outcomes (Ryan and Deci, 2000). These motivational factors, including the feeling of doing meaningful work, responsibility, recognition, and progress, build individual motivation for better workplace performance (Luthans and Doh, 2018). Motivation is a psychological process that provides direction for employee behavior and is linked to internal satisfaction and organizational behavior (Marinak and Gambrell, 2008; Paais and Pattiruhu, 2020).

### Mediating effect of green work environment

The work environment encompasses a broad array of elements including all tools, materials, and methods within a workspace, as well as the surrounding environment that impacts both individual and group dynamics (Sedarmayanti, 2014). It involves factors such as cleanliness, music, and lighting, which collectively shape the workplace’s ambiance (Adiwinata et al., 2022; Kim and Lee, 2022). Beyond these physical factors, a green work environment also includes non-physical elements such as inclusive work structures and supportive leadership, which are crucial for fostering a sustainable work culture (Tian et al., 2020).

A green work environment significantly mediates employee motivation, thereby boosting performance (Al Doghan and Zakariya, 2022). This holistic environment combines both tangible and intangible factors, crafting a workspace that not only supports but also enhances performance by promoting sustainability and well-being. For example, well-lit, clean, and spacious settings help reduce stress and increase mental clarity, thus driving better performance (Majchrzak and Osuch, 2023). Moreover, incorporating green spaces and sustainable practices boosts morale and aligns employee values with organizational goals (Noe et al., 2019; Shahzad et al., 2023).

Furthermore, Green HRM practices integrate critical non-physical elements such as effective leadership and communication (Huo et al., 2022), which play a key role in linking motivation with performance. These elements provide essential structural and emotional support, making employees feel valued and understood, which in turn significantly increases their motivation. Such heightened motivation fosters proactive behaviors toward sustainability and productivity, thereby elevating overall performance metrics (Masri and Jaaron, 2017).

Effective indicators of a well-managed green environment not only encompass amenities and security but also include features that directly influence employee motivation and satisfaction. These environments cultivate a culture of environmental stewardship and enhance a sense of belonging and purpose among employees, crucial motivators for superior performance (Li et al., 2015). By bridging the gap between motivation and performance, a green work environment ensures that employees’ environmental awareness is transformed into productive work outcomes, thus boosting both individual and organizational productivity.

### Mediating effect of employee engagement

Employee engagement is defined as a positive, fulfilling work-related state of mind characterized by vigor, dedication, and absorption (Schaufeli et al., 2002; Saad et al., 2022). It involves a sustained, holistic relationship with work activities, transcending momentary and specific interactions (Kahn, 1990). This engagement becomes a mediator in the relationship between motivation and employee performance, functioning as a critical bridge that translates intrinsic and extrinsic motivations into effective work outcomes. Engaged employees, who are mentally and physically connected to their work (Mustaffa et al., 2022), enhance their performance through high commitment and positive emotions at work (Dessler, 2017).

To foster employee engagement, companies must create optimal conditions, including job challenges, autonomy, variety, feedback, fit, development opportunities, and rewards and recognition (Armstrong and Taylor, 2020). These elements are particularly significant as they directly influence the level of motivation employees feel, which in turn affects their level of engagement. For example, a broad job scope, significant responsibilities, and high workload can strengthen engagement by offering personal achievement and growth opportunities, which are motivating factors.

Autonomy in scheduling work and choosing procedures enhances ownership and control over work outcomes, further motivating employees by giving them a sense of empowerment (Rafique et al., 2022). Meaningful work, coupled with the company’s training and development programs, contributes to growth and fulfillment, thereby maintaining high levels of motivation and engagement (Lin et al., 2021). Rewards and recognition serve as direct motivators that reflect the returns on an individual’s investment of effort and time, enhancing their engagement (Bashir et al., 2023).

Employee engagement encompasses three dimensions: vigor, dedication, and absorption, as identified by Schaufeli et al. (2002). Indicators of engagement include high energy levels and mental resilience at work, willingness to exert effort, and persistence in the face of challenges (Nusraningrum, 2018). By mediating between motivation and performance, engagement ensures that motivated employees translate their enthusiasm into sustained high performance, highlighting its critical role in driving organizational success.

In summary, the hypotheses proposed in this study are as follows:

*H1*: Motivation affects employee performance.

*H2*: Motivation affects a green work environment.

*H3*: Motivation affects employee engagement.

*H4*: Green work environment affects employee performance.

*H5*: Employee engagement affects employee performance.

*H6*: Green work environment mediates the relationship between motivation and employee performance.

*H7*: Employee engagement mediates the relationship between motivation and employee performance.

**Figure 1:**
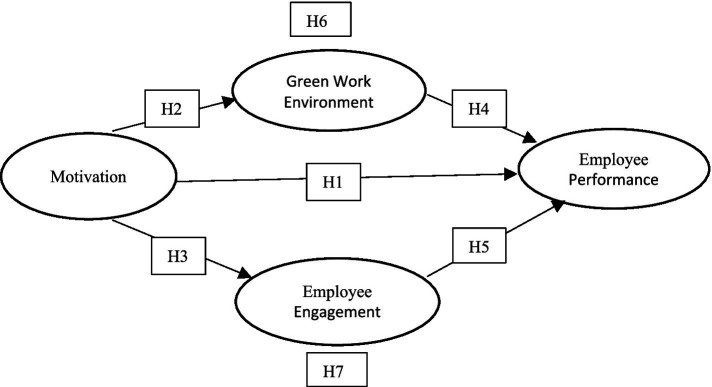
Theoretical model.

The theoretical model of the variables used is made based on the literature review shown in Figure 1.

### Methodology

This study employed a quantitative causal approach through the distribution of both online and offline questionnaires. The population comprises an unspecified number of employees. According to Hair et al. (2017), when using the Structural Equation Modeling (SEM) technique, the sample size should range between 100–200 or be 5–10 times the number of estimated indicators. In this study, the sample size reached 222 logistics employees in Jabodetabek (Greater Jakarta). The questionnaire consisted of 44 items, which are a modification of the original questionnaire, and utilized a 5-point Likert Scale. The constructs measured included employee performance with 10 indicators (adapted from Armstrong and Taylor, 2020), motivation with 14 indicators (adapted from Mathis et al., 2017), employee engagement with 14 indicators (adapted from Dessler, 2017), and a green work environment with 7 indicators (adapted from Sunyoto, 2012).

## Results

The sample is primarily skewed toward males, who make up 62.2% of the sample (138 respondents). In terms of age group, the age groups of 20–30 years and 30–45 years make up significant portions, comprising 41.0% (91 respondents) and 39.2% (87 respondents) respectively. In terms of educational qualification, the sample consists of 59 respondents with an Associate degree, accounting for 26.6% of the total. Additionally, there are 58 respondents with a Bachelor’s degree, representing 26.1% of the sample. The proportion decreases as the educational level increases, with individuals holding a Master’s degree accounting for 11.3% (25 respondents) and those with a Doctoral degree representing 4.5% (10 respondents). Remarkably, 31.5% (70 respondents) of the sample have successfully finished high school. In terms of employment type, it is evident that a significant majority of the respondents, constituting 62.2% of the sample size (138 respondents), are employed in profit-oriented (private) companies. The rest work in state-owned enterprises (12 SOEs) that handle logistics, such as PT Pos and others. The duration of employment experience in the sample varies. Specifically, 15.4% (33 respondents) have <1 year of experience, 46.9% (104 respondents) have between 1 and 5 years of experience, and 38.3% (85 respondents) have more than 5 years of experience. In terms of employment status, it is found that 36.0% of the respondents (80 individuals) are not fully employed, while the majority, 64.0% (142 individuals), are fully employed. The majority of positions within the sample are at the staff level, accounting for 85.6% (190 respondents). There is a smaller representation in supervisory roles (6.3%, 14 respondents) and middle management roles (6.3%, 14 respondents). Only 1.8% of the sample, which corresponds to 4 respondents, occupy top management positions. The sample is geographically divided, with 51.4% (114 respondents) living outside Jakarta and 48.6% (108 respondents) living within Jakarta (Table 1).

**Table 1:** Characteristics of respondents.

Characteristics	Respondent	Frequency	Percentage
Gender	Male	138	62.2
	Female	84	37.8
Age	< 20	14	6.3
	20–30	91	41.0
	30–45	87	39.2
	> 45	30	13.5
Educational qualification	Associate degree	59	26.6
	Bachelor	58	26.1
	Master	25	11.3
	Doctor	10	4.5
	High school	70	31.5
Employment type	Profit oriented (Private)	138	62.2
	Nonprofit oriented (Public)	84	37.8
Length of work	< 1 year	33	15.4
	1–5 years	104	46.9
	> 5 years	85	38.3
Employment status	No	80	36.0
	Yes	142	64.0
Position	Staff	190	85.6
	Supervisor	14	6.3
	Middle manager	14	6.3
	Top manager	4	1.8
Location	Outside Jakarta	114	51.4
	Jakarta	108	48.6

Table 2 indicates that the results of the composite reliability test and Cronbach’s alpha demonstrate satisfactory values. Specifically, all latent variables are deemed reliable since each latent variable’s composite reliability values and Cronbach’s alpha are above 0.70. Consequently, the questionnaire used as a research tool can be considered reliable or consistent.

**Table 2:** Validity and reliability test result.

Variable	Cronbach’s alpha	Rho_A	Composite reliability	Average variance extracted (AVE)
Motivation	0.919	0.700	0.930	0.491
Green work environment	0.856	0.769	0.895	0.591
Employee engagement	0.905	0.718	0.921	0.516
Employee performance	0.882	0.769	0.905	0.489

The collinearity statistics (VIF) test results reveal that the VIF values for item indicators in Motivation, Green Work Environment, Employee Engagement, and Employee Performance are below 5. This suggests that there are no multicollinearity issues.

The R-Square value of the Green Work Environment variable is 0.390, indicating that 39% of its variability can be explained by the variables in the model, namely Motivation. The R-Square value for Employee Engagement is 0.679, meaning 67.9% of its variability is explained by Motivation and Green Work Environment. Employee Performance has an R-Square value of 0.775, suggesting that 77.5% of its variability can be explained by Motivation, Green Work Environment, and Employee Engagement.

Hair et al. (2017) recommends testing the Goodness of Fit of the structural model on the inner model using a predictive relevance value (*Q*^2^) of 0.927775. This implies that 92.7775% of the variation in Employee Engagement and Employee Performance is explained by the independent variables used (motivation and green work environment), indicating the model has relevant predictive value.

Table 3 shows that motivation has a positive and significant effect on the green work environment, as indicated by a beta value of 0.517 (*p* = 0.000). Similarly, motivation positively and significantly affects green work motivation, with a beta value of 0.556 (*p* = 0.000), and employee engagement, with a beta value of 0.556 (*p* = 0.000). Additionally, the green work environment positively and significantly impacts employee performance, with a beta value of 0.173 (*p* = 0.004), Employee engagement also positively and significantly affects employee performance, as indicated by a beta value of 0.277 (*p* = 0.005). Additionally, Table 3 shows the significant indirect effect of motivation on employee performance through green work motivation (beta = 0.125, *p* = 0.000) and employee engagement (beta = 0.08, *p* = 0.008). All research hypotheses have been tested and confirmed to demonstrate a significant effect.

**Table 3:** Hypothesis testing results.

Hypothesis	Path coefficient	*T* statistics	*p* values	Decision
*H1*. Motivation ➔ Employee Performance	0.517	6.043	0.000	Accepted
*H2*. Motivation ➔ Green Work Environment	0.624	7.312	0.000	Accepted
*H3*. Motivation ➔ Employee Engagement	0.556	7.695	0.000	Accepted
*H4*. Green Work Environment ➔ Employee Performance	0.173	2.866	0.004	Accepted
*H5*. Employee Engagement ➔ Performance	0.277	2.839	0.005	Accepted
*H6*. Motivation ➔ Green Work Motivation ➔ Employee Performance	0.125	3.678	0.000	Accepted
*H7*. Motivation ➔ Employee Engagement ➔ Employee Performance	0.080	2.234	0.008	Accepted

## Discussion

Motivation exerts a positive and significant influence on a green work environment, indicating that when employees are highly motivated, they contribute to creating a more environmentally friendly and pleasant workplace (Noe et al., 2019; Shahzad et al., 2023). This relationship is crucial, as a green work environment encompasses not only the physical aspects but also considers social and economic influences that bear upon sustainability (Masri and Jaaron, 2017). Employee motivation is instrumental in fostering a culture of environmental stewardship, encouraging employees to actively participate in maintaining and protecting their surroundings (Li et al., 2015). The research underscores the role of employee motivation in initiating proactive behaviors toward creating an environmentally sustainable work environment.

The impact of motivation extends to employee performance, where a positive correlation indicates that heightened motivation among employees leads to increased performance levels (Ryan and Deci, 2000; Danish et al., 2015). This relationship is supported by previous studies which found a significant and dominant influence of motivation on employee performance, making it a critical variable in the overall performance matrix (Mulyani et al., 2019). Theoretically, this relationship is framed within the context of path models that link motivation to engagement and subsequently to performance (Ito and Umemoto, 2023). The influence of motivation on engagement has been conceptualized in prior research as a causal link, represented in theories such as self-determination (Ryan and Deci, 2017) and the expectancy-value theory of achievement motivation (Wigfield, 1994). These theories collectively emphasize the pivotal role of motivation in shaping employee attitudes and behaviors toward their roles and responsibilities within the organization.

Moreover, motivation significantly affects employee engagement, suggesting that higher levels of motivation among employees are likely to enhance their engagement with their work. This strong influence of motivation on employee engagement is critical in creating a positive work atmosphere, where employees are happy and more involved in their tasks (Schaufeli, 2013; Putra et al., 2017). This increase in employee involvement often occurs subtly, but it significantly contributes to organizational sustainability and growth (Sharma and Singh, 2016). External motivational factors also exhibit a positive relationship with work engagement, encompassing both positive motivations (such as rewards and incentives) and negative motivations (like sanctions and punishments) (Nusraningrum and Dwi, 2018; Cimbaljević et al., 2020). This bifurcation of motivation into positive and negative external factors highlights the complexity of motivational dynamics in influencing employee engagement (Christian et al., 2011; Masvaure and Maharaj, 2014).

Furthermore, a green work environment positively and significantly influences employee performance. A work environment that is supportive and environmentally conscious is found to bolster employee performance (Buil et al., 2019; Kim et al., 2019). This finding is particularly relevant in the context of rising concerns about environmental degradation due to industrial activities. The congruence between individual values and the work environment, such as job alignment, organizational culture, and relationships with managers and co-workers, facilitates behaviors that align with personal and organizational environmental goals.

The research also reveals that a green work environment significantly enhances employee engagement. When employees operate in a green work environment, they tend to show higher levels of dedication and involvement in their work, characterized by feelings of significance, enthusiasm, and challenge (Schaufeli, 2013). Recent studies have combined the concept of a green work environment with employee engagement, emphasizing the need for management to continue investing in environmental sustainability initiatives. Such initiatives reassure employees of the organization’s commitment to environmental preservation, thereby fostering a culture of engagement and productivity (Karatepe et al., 2022).

Lastly, employee engagement is shown to have a positive and substantial effect on employee performance. Engaged employees are more likely to exhibit improved performance, and feel a sense of belonging and ownership within the organization (Nazir and Islam, 2017; Carter et al., 2018; Gustiah and Nurhayati, 2022). Jobs that require a variety of activities or skills, coupled with clear organizational goals and communication, tend to result in higher levels of employee engagement.

This research implies that logistics companies can enhance employee performance, characterized by improved output quality, efficiency, and effectiveness, through the cultivation of a green work environment and by fostering employee motivation and engagement. These factors are pivotal in shaping employee attitudes such as teamwork, creativity, and a proactive approach to their work (Nusraningrum et al., 2023b). Employee performance indicators consisting of quantity of work, quality of results, time regularity, cost-effectiveness, safety, attendance, compliance with policies, initiatives and innovations, teamwork, and customer focus, form a holistic evaluation framework. The quantity of work and the quality of results reflect productivity and customer satisfaction, while time regularity and cost-effectiveness describe operational efficiency. Safety and attendance indicate social responsibility and consistency, while policy adherence indicates adherence to the corporate structure. Initiative and innovation stimulate development and adaptation, while teamwork and customer focus strengthen the company’s internal and external relationships. A holistic analysis of these ten indicators helps in assessing overall employee performance and promoting sustainable growth and excellence in an organization (Armstrong and Taylor, 2020).

### Conclusion, limitations, and recommendation

The hypothesis testing results suggest that employee performance in the logistics companies under study is significantly influenced by factors such as motivation, employee involvement, and a green work environment. This highlights the critical importance of fostering employee motivation to actively engage in organizational activities and support a sustainable green work environment, ultimately leading to enhanced employee performance. These findings underscore the significance of human resource management practices as an integral part of the company’s overall operations management strategy. The study advocates for policies prioritizing proactive employee attitudes, enhancing workplace security, and bolstering enthusiasm for work to improve employee performance.

However, the study is geographically limited to Jakarta and its surrounding areas, which may not fully represent other regions or countries, potentially affecting the generalizability of our results. The sample composition, which is predominantly male and comes from the private sector, raises additional concerns about the study’s representativeness. Furthermore, the data collection instruments were modified questionnaires designed specifically for the Jakarta logistics sector, necessitating detailed accounts of adaptation and validation to improve methodological soundness.

To address these limitations, future research should aim for a broader geographical scope that includes more diverse regions or countries, as well as a gender-balanced sample of public sector employees. Further research could provide more detailed information on how data collection instruments are adapted and validated for specific sectors, as well as investigate other potential influences on employee performance, such as technological advancement, leadership styles, and organizational culture. These efforts would provide a more in-depth understanding of the dynamics between the variables under consideration, as well as improve the generalizability and methodological rigor of the research. The green work environment section, in particular, should include more comprehensive research on green activities related to organizational policies on work motivation and employee engagement to provide a more in-depth examination of these critical factors.

In conclusion, while this study provides useful insights into the factors that influence employee performance in logistics companies, it also emphasizes the importance of ongoing research to improve our understanding and application of human resource management practices in improving organizational performance.

## Data availability statement

The raw data supporting the conclusions of this article will be made available by the authors, without undue reservation.

## Ethics statement

Ethical approval was not required for the studies involving humans because the study was conducted according to the guidelines of the Declaration of Helsinki and following academic ethics. The studies were conducted in accordance with the local legislation and institutional requirements. The participants provided their written informed consent to participate in this study.

## Author contributions

DN: Conceptualization, Data curation, Formal analysis, Investigation, Methodology, Project administration, Resources, Software, Supervision, Validation, Visualization, Writing – original draft. AR: Conceptualization, Data curation, Formal analysis, Investigation, Methodology, Resources, Software, Validation, Visualization, Writing – review & editing. WW: Conceptualization, Funding acquisition, Methodology, Visualization, Writing – review & editing. LJ: Writing – review & editing. LU: Funding acquisition, Writing – review & editing.
